# Supplementary material for: Preparation of Monoclonal Antibody against Pyrene and Benzo [a]pyrene and Development of Enzyme-Linked Immunosorbent Assay for Fish, Shrimp and Crab Samples
Source: Foods. 2022 Oct 15;11(20):3220. doi: 10.3390/foods11203220 (PMC9602326; doi:10.3390/foods11203220)
Supplement: Supplementary file 1 [file foods-11-03220-s001.zip › foods-1914515-supplementary.pdf]

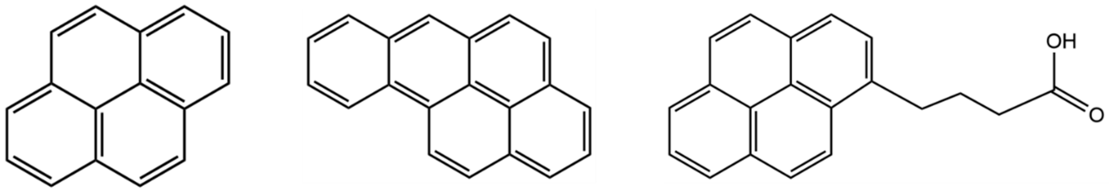

PYR BaP PBA

Figure S1 Structure of PYR, BaP and PBA

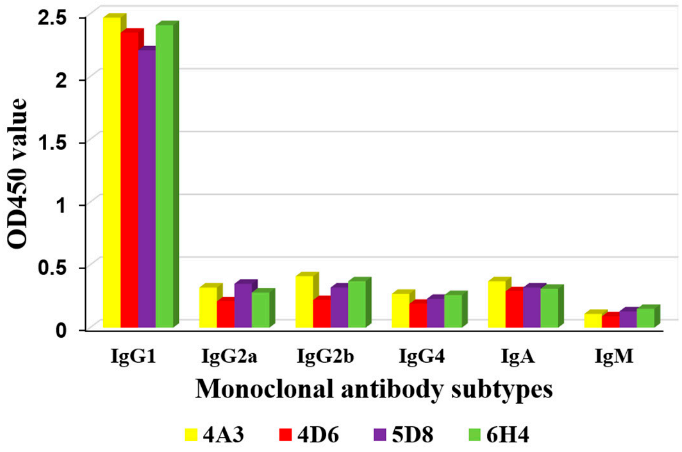

Figure S2. Subtype determination of four monoclonal antibodies

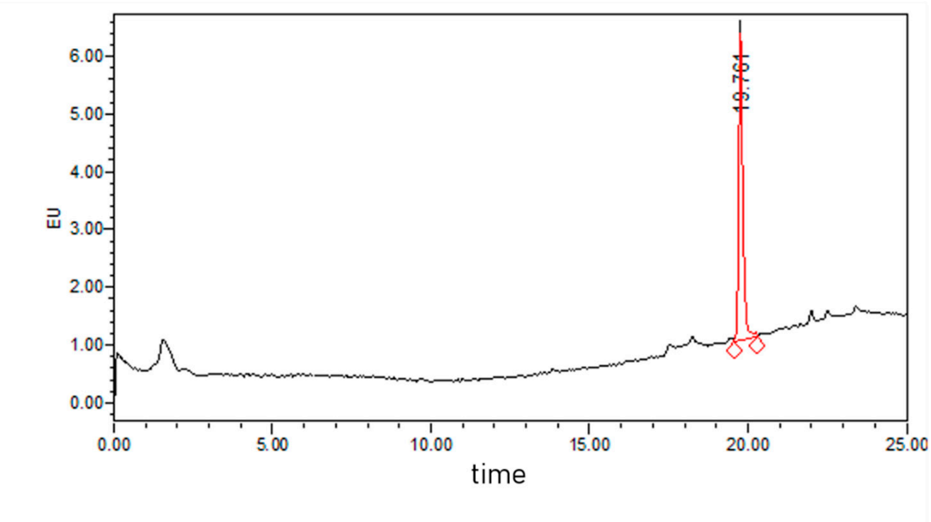

Figure S3 The liquid chromatogram spectrum of PYR in fish sample (10 ng/mL)

Table S1 Comparison of absorbance and inhibition rate of antigens with different coupling ratio on immunized mice

immunogen	diluted ratio of serum(1:x)	coating antigen (blank hole OD_{450} value)			Inhibition rate（B/B_0）		
		**B_1**	**B_2**	**B_3**	**B_1**	**B_2**	**B_3**
**A_1**	1:3200	1.034±0.026	1.381±0.023	1.576±0.035	0.57	0.52	0.45
**A_2**	1:6400	1.487±0.043	1.765±0.017	1.922±0.028	0.47	0.42	0.36
**A_3**	1:12800	1.642±0.021	1.956±0.040	2.127±0.052	0.39	0.28	0.21

Note: B: OD_{450} nm value of wells containing PYR standard; B_0: the OD_{450} nm value of the wells that contained only assay buffer. Inhibition rates were calculated at the same drug concentration.

Table S2. Dilutions and IC_{50} values of four monoclonal antibodies

Antibody	4A3	4D6	5D8	6H4
Antibody dilution (1:X)	20000	20000	16000	20000
IC_{50} value(μg/L)	6.2	5.3	5.9	6.6

Table S3 Optimization of ELISA conditions

**Optimization conditions**	**Parameter value**	**Blank hole OD_{450} value**	**IC_{50}(μg/L)**
Coating concentration (μg/mL)	1	1.562±0.014	5.7±0.14
	2	2.001±0.021	4.2±0.11
	4	2.124±0.023	4.9±0.23
	8	2.201±0.047	6.2±0.15
The diluted concentration of mAb (1: X)	10000	2.371±0.051	6.7±0.21
	15000	2.187±0.044	5.8±0.23
	20000	2.068±0.035	4.4±0.14
	25000	1.847±0.021	5.2±0.31
Incubation time of primary antibody (min)	25	1.897±0.032	4.9±0.26
	35	2.003±0.018	3.7±0.22
	45	2.103±0.027	4.7±0.16
	55	2.116±0.037	5.3±0.20
Incubation time of HRP-IgG (min)	30	1.902±0.022	4.9±0.33
	40	2.032±0.035	4.1±0.18
	50	2.133±0.037	5.6±0.15
	60	2.157±0.039	6.1±0.17
Working concentration of HRP-IgG (1:X)	5000	2.268±0032	5.6±0.27
	6000	2.037±0.028	4.3±0.21
	7000	1.832±0.035	4.9±0.18
	8000	1.597±0.033	4.6±0.14

Table S4 Cross-reactivity of 4D6 mAb with 16 polycyclic aromatic hydrocarbon drugs

Drugs	IC_{50} (μg/L)	cross-reactivity (%)	R^2
Pyrene	3.73	100	0.9975
Benzo[a]pyrene	9.71	38	0.9975
Fluoranthene	45.83	8	0.9972
Naphthalene	＞373	＜1	
Acenaphthene	＞373	＜1	
Acenaphthylene	＞373	＜1	
Fluorene	＞373	＜1	
Phenanthrene	＞373	＜1	
Anthracene	＞373	＜1	
Benz[a]anthracene	＞373	＜1	
Chrysene	＞373	＜1	
Benzo[b]fluoranthene	＞373	＜1	
Benzo[k]fluoranthene	＞373	＜1	
Dibenz[a,h]anthracene	＞373	＜1	
Benzo[g,h,i]perylene	＞373	＜1	
Indeno[1,2,3-cd] pyrene	＞373	＜1	
